# Advancing Health Equity Through Substance Use Medical Record Data Sharing: Insights from Healthcare Providers

**DOI:** 10.3390/ijerph22040462

**Published:** 2025-03-21

**Authors:** Mengyi Wei, Anita Murcko, Sai Prathyusha Nookala, Dharma Teja Bhattu, Sai Jahnavi Vemula, Darwyn Chern, Eric Lott, Mary Jo Whitfield, Nick Stavros, Deborah Ariosto, Maria Adela Grando

**Affiliations:** 1 College of Health Solutions, Arizona State University, Phoenix, AZ 85004, USA; mwei32@asu.edu (M.W.); anita.murcko@asu.edu (A.M.); snookal4@asu.edu (S.P.N.); dbhattu@asu.edu (D.T.B.); svemul28@asu.edu (S.J.V.); deb.ariosto@gmail.com (D.A.); 2 Copa Health, Mesa, AZ 85205, USA; darwyn.chern@copahealth.org; 3 Community Bridges Inc., Phoenix, AZ 85034, USA; elott@cbridges.com; 4 Jewish Family and Children’s Services, Phoenix, AZ 85037, USA; maryjo.whitfield@jfcsaz.org; 5 Community Medical Services, Phoenix, AZ 85021, USA; nick.stavros@cmsgiveshope.com

**Keywords:** HEDIS metrics, substance use disorder, data sharing

## Abstract

Background: Better care is delivered when patients and providers share health information. Unfortunately, critical health data are often unavailable due to fragmentation within healthcare systems. Sensitive health information, like substance use disorder, is often sequestered in ways that do not meet patient data privacy choices and provider data access needs. This study explored healthcare providers’ perspectives on barriers and facilitators to substance use data sharing and its impact on care. Methods: Focus groups were conducted with 31 healthcare providers from four treatment facilities. Discussions focused on privacy concerns, data-sharing workflows, and scenarios involving four Healthcare Effectiveness Data and Information Set (HEDIS) substance use disorder specific metrics. Open coding identified key concepts, and thematic analysis was employed to identify barriers and facilitators influencing data sharing and care outcomes. Results: Providers identified five main barriers: patient reluctance to share (48%), data access challenges (42%), poor provider coordination (29%), incomplete health information (26%), and complexity of privacy regulations (23%). Key facilitators included patient understanding (26%), patient–provider relationship (16%), and reliability of health information systems (16%). Discussion: This study sets the stage for understanding and addressing sensitive healthcare data access and privacy concerns through improved care coordination, systems interoperability, education, and policy reform.

## 1. Introduction

Substance use disorders (SUDs) present a significant challenge to healthcare systems, particularly in advancing health equity and ensuring timely, integrated care for underserved populations [1,2]. Stigma associated with SUD limits access to high-quality treatment, affecting marginalized communities and exacerbating systemic inequities [3,4]. These barriers impede care engagement, delay treatment initiation, and lower the likelihood of sustained recovery. Addressing them requires effective data-sharing practices to support coordinated, patient-centered care and meet care performance measures, such as the Healthcare Effectiveness Data and Information Set (HEDIS) [5,6,7].

HEDIS is a widely used set of performance metrics designed to evaluate the quality of care provided by health plans and healthcare organizations. Developed by the National Committee for Quality Assurance (NCQA), it assesses healthcare effectiveness across insurance plans, providers, and population health programs, influencing quality improvement and policy decisions [8]. It includes over 90 measures, 13 of which focus on behavioral health, with 4 specifically assessing opioid use disorder (OUD) [8]. For SUD care, metrics such as Initiation and Engagement of SUD Treatment (IET), Follow-Up After Emergency Department Visit for Substance Use (FUA), and Follow-Up After High-Intensity Care for SUD (FUI) help to ensure equitable access to treatment, timely follow-up, and effective care coordination [9,10,11]. Compliance with HEDIS standards aligns with best practices in quality care, ensuring that individuals with SUD receive timely, evidence-based treatment. By tracking these key performance measures, HEDIS helps promote early intervention, continuity of care, and provider accountability, ultimately improving patient engagement and treatment outcomes [5,10]. However, achieving compliance with HEDIS standards often faces systemic obstacles, including legal and regulatory restrictions, fragmented health information systems, and sociocultural stigma [3,12,13].

Moreover, integrating SUD care into general medical practice is increasingly recognized as a critical pathway to combatting the opioid epidemic by expanding access and improving care, yet effective data sharing remains a persistent challenge [1,14]. Many providers emphasize the need for better access to SUD-related data, especially within integrated care models [15,16]. Studies involving stakeholders in Oregon and the National Drug Abuse Treatment Clinical Trials Network (CTN) highlight that existing data-sharing policies create legal ambiguity, which disrupts communication across providers. Longstanding protected health data regulations, like the Health Insurance Portability and Accountability Act of 1996 (HIPAA) and, more recently, those specific to restricting substance use disorder disclosures like 42 CFR Part 2, often hinder the information flow necessary for comprehensive care [17,18,19]. To navigate these barriers, healthcare organizations rely on manual processes that are time-consuming and error-prone [10,11].

Adding to these challenges is the stigma surrounding SUD [3]. Providers often face a conflict between the need to share comprehensive patient information to ensure safe and effective treatment and the obligation to protect patient privacy and prevent stigma, particularly under the constraints of 42 CFR Part 2 [13]. Compounding this issue, societal stigma and discrimination further discourage individuals with SUD from seeking care or sharing critical information [20]. This reluctance impacts data accuracy and care coordination, affecting marginalized populations burdened by systemic inequities.

As revealed by Goal et al. in a scoping review of SUD HEDIS metrics, only one of the 28 included studies utilized interviews, with the majority relying on retrospective cohort designs or randomized controlled trials [21]. Yarborough et al. conducted key informant interviews in a study that examined patient and system characteristics related to HEDIS measures of Alcohol and Other Drug Treatment Initiation and Engagement but did not explore providers’ perspectives on data sharing [22]. To fill this gap, our study seeks to identify key barriers and facilitators influencing compliance with HEDIS SUD metrics, offering strategies to improve data-sharing practices and enhance the delivery of timely, coordinated, and equitable care.

## 2. Materials and Methods

### 2.1. Study Design

We conducted focus groups to examine healthcare providers’ perceptions of SUD data sharing and their experiences with HEDIS metrics. The Arizona State University Institutional Review Board (IRB) approved this study (STUDY00016171). The participants provided informed, written consent before participating in the focus groups.

### 2.2. Recruitment

We recruited healthcare professionals involved in SUD-data-sharing processes from four sites: two integrated primary care and behavioral health clinics that had participated in a previous project and two addiction treatment clinics selected to expand geographic representation and diversity in care facility types. Eligible participants included behavioral health professionals actively engaged in SUD data sharing, such as psychiatrists, advanced-practice providers (e.g., nurse practitioners, physician assistants), social workers, clinical psychologists, therapists, certified SUD counselors, and psychiatric nurses. Excluded individuals were not behavioral health professionals or were not involved in SUD-data-sharing processes.

We recruited participants through site leads. We aimed to enroll 7 to 8 participants per site (28 to 32 participants in total). We provided interested individuals with an eligibility survey, which gathered information regarding their professional role, years of experience with SUD data sharing, organizational affiliation, and availability for focus group sessions. We contacted those who met the eligibility criteria with additional details about the focus group sessions.

### 2.3. Selection of HEDIS Metrics

We distributed a survey to research sites and team members to assess the perceived relevance of nine 2025 HEDIS metrics to SUD data sharing [8]. Based on 13 responses, we identified the following four metrics as most pertinent, and we selected them for focus group discussions:

1. Follow-Up After Emergency Department Visit for Substance Use (FUA): This measures the percentage of emergency department (ED) visits for substance use that are followed by a follow-up visit within 7 and 30 days.
2. Initiation and Engagement of Substance Use Disorder Treatment (IET): This evaluates the percentage of patients diagnosed with a substance use disorder who initiate treatment within 14 days of diagnosis and engage in additional treatment sessions within 34 days.
3. Follow-Up After High-Intensity Care for Substance Use Disorder Within 7 Days (FUI): This tracks the percentage of patients discharged from high-intensity care settings, such as inpatient or residential treatment programs, who receive follow-up care within 7 days.
4. Use of Opioids from Multiple Providers (UOP): This monitors the percentage of patients receiving opioids from multiple prescribers and pharmacies during the measurement period.

### 2.4. Provider Focus Group

Each Zoom session accommodated up to six participants and lasted 90 to 120 min. Participants were assigned with a unique identifier throughout the session, to maintain confidentiality and encourage open discussion. The selection of focus group topics was guided by two key investigative threads: (1) assessing the potential impact of enhanced SUD data-sharing strategies on care outcomes, such as timely follow-up, using HEDIS metrics, and (2) identifying challenges in acute care discharge and follow-up processes from the perspective of frontline clinic staff to inform data-sharing improvements. Specifically, the sessions focused on core topics related to scenarios involving selected HEDIS metrics—IET, FUA, FUI, and IET—and included discussions on the challenges and facilitators associated with accessing and sharing SUD data, perceived patient attitudes toward data privacy, and the impact of regulations such as HIPAA and 42 CFR Part 2.

A lead moderator guided the conversations, encouraged open discussion, and kept participants on track, while two co-moderators took notes and ensured the accuracy of the recorded data. We recorded and transcribed the sessions using Zoom Workplace Version 6.3.11.

### 2.5. Data Analysis and Trustworthiness

We analyzed the focus group data to identify key barriers and facilitators to SUD data sharing. We conducted open coding, systematically reviewing transcripts to extract challenges and enabling factors mentioned by participants [23]. We broke the data into smaller, meaningful segments that reflected participants’ experiences. Next, we used axial coding to group related codes into broader categories, such as “stigma and legal fears” and “data fragmentation”.

We linked these findings to overarching themes, such as perceived barriers and factors that facilitated effective data sharing [23]. Finally, we conducted a thematic analysis to refine these insights into overarching themes, offering a structured understanding of the barriers and facilitators participants encountered in sharing SUD data [23]. We used the MAXQDA software version 24 to manage and analyze the focus group data [24].

We ensured the rigor and reliability of the findings with several strategies to enhance trustworthiness. Two researchers analyzed the themes to incorporate diverse perspectives and reduce bias. In addition, a physician reviewed and helped to refine thematic interpretations, while negative case analysis validated findings by identifying and examining conflicting data for a balanced understanding [23,25].

## 3. Results

### 3.1. Participants and HEDIS Experience

A total of 31 participants completed the focus groups, which lasted an average of 90 min each and were conducted across 11 sessions at four clinical sites. More than half (n = 19) of the participants were female. The majority (n = 19) reported having more than five years of experience working in SUD care. The participants represented diverse professional roles, including counselors (n = 8), advanced practitioners (n = 7), nurses (n = 3), directors/co-directors (n = 3), psychiatrists (n = 2), addiction medicine fellows (n = 2), clinical managers (n = 2), clinicians (n = 2), an assertive community treatment (ACT) specialist (n = 1), and a case manager (n = 1). Based on the HEDIS familiarity responses, 35% (n = 11) of participants reported they had “Never” heard of HEDIS, 55% (n = 17) indicated they had “Heard” of it, and 10% (n = 3) described their familiarity as “Very”.

### 3.2. Barriers

Participants identified five barriers during the focus groups: (1) patient reluctance to share; (2) data access challenges; (3) poor provider coordination; (4) incomplete health information; and (5) complexity of SUD laws (Table 1).

#### 3.2.1. Patient Reluctance to Share Due to Stigma and Legal Concerns

Patients are often reluctant to share their SUD information due to fear of stigma and judgment and legal concerns, both of which impede effective data collection and sharing. This barrier was discussed by 48% of focus group participants.

Participants emphasized that stigma and provider judgment significantly hinder patients’ willingness to disclose SUD data, impacting both treatment and broader aspects of their lives. One provider explained, “I think the biggest is still that stigma. The stigma in the community makes them afraid…” Beyond the clinical environment, one provider shared that patients are reluctant to disclose their SUD diagnosis due to concerns about the broader impact on their lives. They explained that it “can impact people’s housing… family situations, relationships”.

In addition, patients involved in legal processes, such as child custody cases, may worry that sharing their SUD diagnosis could negatively affect their case, causing them to withhold vital information. They noted, “It’s really based off of other things going on like… DCS (Department of Child Safety) or other legal issues that they may have going on, that they’re more concerned about than per se health concerns”.

Similarly, another provider highlighted that legal fears limit patients’ willingness to share the SUD data, particularly when involved in sensitive legal situations. For example, patients may worry, “Will it impact my job or affect child custody or divorce proceedings?” For those on criminal probation, the concern was even more pressing: “Will any of this get back to my probation officer, and could it end with me back in jail or prison?”

#### 3.2.2. Data Access Challenges from External Facilities

Providers reported significant barriers to accessing timely and complete data from external care facilities, especially correctional facilities and hospitals, leading to disruptions in continuity of care, particularly for patients requiring medication-assisted treatments (MATs). This barrier was discussed by 42% of focus group participants.

One participant described the challenge of coordinating buprenorphine dosing due to the lack of direct information sharing between jail healthcare settings and external clinics: “We had a very hard time and had to delay someone’s buprenorphine dosing by one day”, they explained, as they struggled to obtain records from another clinic that had managed the medication during the patient’s incarceration. Another provider shared the difficulties that arose when patients were discharged from jails without notice, making it “extremely difficult to catch them”. They explained, “We need proof that they have been on Sublocade for the insurance to approve the long-acting injection, it’s like a police chase”.

Additionally, one provider highlighted the challenge of obtaining hospital records, stating, “I think that’s probably the hardest. I think that’s the hospital records”. Another participant noted similar difficulties with accessing “anything that’s external”, such as lab reports or hospital discharge paperwork, particularly for intake or transfer cases. They explained, “Sometimes somebody will send over a chart that’s like thousands of pages long, and it’s all one PDF, so it can take some time to sort through and organize…”

#### 3.2.3. Poor Provider Coordination

Providers often encountered poor coordination between Medication for Opioid Use Disorder (MOUD) and medication-assisted treatment (MAT) clinics and mental health agencies, which complicates transitions for patients moving between care settings. When information was not shared promptly, providers were forced to make decisions with incomplete data, leading to potential treatment delays or medication errors. This barrier was discussed by 29% of focus group participants.

One provider described difficulties during patient transfers: “A client was transferring from another MOUD clinic… If our nurses are trying to call that place to confirm… and no one’s answering the phone, unfortunately, the client will be starting off at 30 milligrams, which is a lot lower than what they’re taking on a daily basis”. Another provider noted how incomplete documentation disrupts treatment: “When clients transfer from other agencies… not having that information can impact dosage and take-homes, sometimes requiring patients to start over”.

In some cases, this lack of coordination extends to other mental health programs, leaving providers reliant on patient-reported information without access to formal treatment records. “When it comes to their connection with any another agency, whether it’s an IOP (Intensive Outpatient Program), a PHP (Partial Hospitalization Program), and… an MAT Clinic… I do have to go based off what the client says, but I can’t see… any other specific notes from that person who ran the groups or anything”.

#### 3.2.4. Incomplete and Outdated Health Information

Participants noted that health information systems, including Health Information Exchanges (HIE), Prescription Drug Monitoring Programs (PDMPs), and methadone management software [26], often lack comprehensive and up-to-date patient information. This barrier was discussed by 23% of focus group participants.

A lack of interoperability is a significant issue, particularly in clinics that rely on clinic-selected methadone management software as their primary data management tool. Providers frequently face challenges navigating and accessing information from these systems. One participant described the issue, stating, “The program we use is not great… everything seems hard to find or in one place. It’s very like having to look around at a lot of different places to find all the information”. Another provider echoed this frustration: “There’s no exchange with Methasoft and other electronic prescribing records that I’m aware of”.

Technical issues with HIE pose challenges for providers attempting to access timely patient information. One provider described recent difficulties: “The last 4 days, there’s something wrong with the HIE data… I normally use it to find out who’s been hospitalized, and for some reason, that stuff hasn’t been coming across”.

Additionally, statutory limitations in PDMP contribute to incomplete records, particularly with methadone reporting. Methadone programs only “administer” and “dispense” methadone and buprenorphine. As such, if a patient receives treatment at an Opioid Treatment Program (OTP), it is not reported to state PDMPs due to protections under 42 CFR Part 2. As one provider noted, “When someone comes in, and they’re going to another OTP, Methadone does not get recorded on their PDMP… it can be a major safety issue if those patients go to primary care, and the primary care doesn’t know that the patient’s taking Methadone… that’s an interaction with the benzo that I was gonna give them, or you know it can be, it can be an issue”.

#### 3.2.5. Complexity of Privacy SUD Laws

The complexity of federal and state privacy laws, particularly 42 CFR Part 2 and HIPAA, pose a significant barrier to sharing SUD data among healthcare providers. This barrier was discussed by 23% of focus group participants.

Although these regulations are designed to protect patient privacy, they often hinder the sharing of essential information necessary for coordinated care. One provider explained, “I think specifically to medications for opioid use disorders, state regulations kind of tie our hands sometimes when we have certain information we can’t share. There’s no wiggle room”. Another participant highlighted confusion during data transfers: “42 CFR Part 2 can cause a lot of confusion and nervousness among many clinics, especially in data transfers between non-SUD settings and SUD settings… In a primary care clinic, they might not be at all familiar, so they might send over records they didn’t have permission to send because they thought they had it filled out correctly”.

While these laws aim to protect patients, they unintentionally obstruct care. One provider described them as a “double-edged sword”, explaining that without patient consent, providers often rely on “subtleties… that maybe we wouldn’t have to if there were open lines of communication”. The volume of paperwork that must be completed during clinic intake processes further complicates compliance. A participant noted, “The intake process can take one to 3 h, and they might sign 10 to 20 forms”.

Participants also highlighted the severe consequences of violating these laws, which contribute to reluctance to access or share SUD data. One provider noted, “Nobody wants to be accused of violating HIPPA or 42 CFR. Which are the regulations that regulate patient privacy and data. So, I think that’s still a challenge”. Another provider elaborated, “The penalties for violating HIPPA, or 42 CFR. Part 2 are fairly heavy, both for the organization as well as a particular individual, particularly if they’re licensed”.

### 3.3. Facilitators

Participants identified three facilitators during the focus groups: a trusting patient–provider relationship enhances willingness to share SUD data, patient understanding of reason for sharing reduces reluctance to disclose SUD data, and availability of health information systems that support SUD data access and coordination (Table 1).

#### 3.3.1. Patient–Provider Relationship Enhances SUD Data Sharing

A trusting patient–provider relationship enhances patients’ willingness to share sensitive SUD data, as they feel assured that their information will be used to support their care and handled responsibly. This facilitator was discussed by 16% of focus group participants.

One provider explained the importance of trust and communication as a foundation for open disclosure. “I think the most important [thing is] for the patient and the provider to have trust… so the patient feels that [they have] trust in the provider and can… have very good communication. After you get that trust from the patient, they can easily… share their information”.

#### 3.3.2. Patient Understanding Reduces Reluctance to Share SUD Data

Providing patients with clear explanations about how their SUD data will be used can reduce their anxiety and reluctance to share, as they become more comfortable with the role their information plays in their treatment. When patients understand that sharing their data supports their safety and care, they are more likely to consent. This facilitator was discussed by 26% of focus group participants.

One provider stressed the importance of explaining the purpose behind data sharing: “Having open lines of communication is going to be better for everyone… just trying to get them to understand why we’re asking for it and that it’s not punitive or we’re trying to take something away. So, we’re just trying to maximize safety. So, I’d say the rule of thumb is to involve, explain it, and then get their buy-in”.

Another provider emphasized that patients should be informed about the importance of sharing their SUD history with primary and external care providers, particularly for medication safety. “I just think it’s important to be able to let our clients know… that their SUD history… is important to share with primary care and external care outside of here because medication can really… cause something bad to happen”, they noted.

#### 3.3.3. Health Information Systems Supporting SUD Data Access and Coordination

Health information exchange (HIE) and PDMP were identified as crucial in supporting data sharing for SUD treatment, enabling providers timely access essential patient information. This facilitator was discussed by 16% of focus group participants.

One provider illustrated the value of specific data access through HIE, explaining, “If we can get to some information from the HIE, that will be very helpful for us… like a UDS (urine drug screen) screen, or how they presented, or what was the treatment they gave”. As another participant described, the ease of access offered by HIE “would make life so much easier for the providers or for the care providers out in the outpatient setting”.

In addition to HIE, PDMP is crucial for monitoring prescription patterns and identifying potential risks, especially when patients see multiple providers. One participant shared, “I definitely use that PDMP… even someone getting an opiate from one prescriber and a benzo from another prescriber… will raise a flag for me”.

## 4. Discussion

This is the first study investigating barriers and facilitators regarding SUD data sharing from the provider perspective in the context of SUD HEDIS metrics scenarios. We note that while the participants acknowledge a priority of delivering care that adheres to generally accepted guidelines and most had heard the term “HEDIS”, they were largely unaware of the specific metrics applied to SUD care delivery, including those used to anchor the focus group cares discussion. Therefore, our providers were primarily focused on the care gaps their patients experienced due to limited health data access, rather than on HEDIS performance metrics. This emphasis on barriers reflects the providers’ firsthand experiences and the pressing need for solutions to improve data accessibility and care delivery.

Our findings underscore the need for both policy reforms with regulatory guidelines that balance patient privacy with effective care coordination and supportive, interoperable technology to improve health data information sharing for SUD care. Five main barriers and three key facilitators were identified by front-line providers delivering SUD care.

Barriers such as stigma and legal concerns were most frequently discussed, with nearly half of participants emphasizing their role in amplifying disparities. This study highlights how stigma hinder equitable care delivery, aligning with prior research showing that patients’ fears of judgment and provider-based stigma reduce their willingness to share sensitive SUD-related information [27,28,29]. Stigma also creates an environment where providers may hesitate to discuss SUD-related needs openly, exacerbating care inequities, but targeted communication training can foster trust, encourage disclosure, and enhance care quality [30]. Additionally, many fear that disclosing SUD data could lead to legal repercussions, such as criminal charges, loss of employment, or jeopardized child custody [18].

Data access challenges, especially when interacting with external institutions, such as correctional facilities or hospitals, emerged as a significant barrier. Providers highlighted delays and incomplete information, including difficulties in obtaining accurate medication histories for patients transitioning between facilities, significantly disrupting continuity of care. These disruptions were particularly harmful to patients receiving MAT, where timely adjustments are essential for effective outcomes [31].

Poor provider care coordination further compounded these issues. Transitioning between clinics or agencies frequently resulted in incomplete or delayed documentation, leading to errors or treatment delays [32]. This reliance on patient-reported information, which is often inaccurate or incomplete, reveals structural gaps that undermine equitable care delivery.

Health information systems, such as the HIE, EHR, and PDMP, emerged as both barriers and facilitators. While these systems offer potential solutions for seamless care coordination, their inconsistent functionality, incomplete data, and limited interoperability, even amongst different sites of the same organization, exacerbate existing disparities. While these findings align with prior research on access to health data, they specifically illuminate how these limitations disrupt SUD care, such as errors in medication adjustments or missed opportunities for timely treatment [33].

Three facilitators emerged as critical to promoting equitable data sharing. Education for patients and providers was a central theme. When patients understand how their information will be used to enhance care and ensure safety, they are more likely to consent to data sharing. Transparent communication and clear explanations were effective strategies for building patient confidence, ultimately fostering equitable engagement with healthcare systems [34]. Provider education is essential, particularly in behavioral health, addiction, and physical health settings. The complexity and ambiguity of current policies, such as 42 CFR Part 2, underscore the need for targeted training to help providers navigate data-sharing regulations confidently while ensuring compliance and patient privacy.

A trusting relationship between providers and patients was another important facilitator. Consistent with previous research, trust was identified as a cornerstone for reducing stigma and encouraging data sharing [35]. Providers emphasized the importance of rapport-building, particularly in underserved populations, where trust deficit is often a barrier to care.

Finally, optimized health information systems, including HIEs, EHRs, and PDMPs, were highlighted as potential enablers of equitable care coordination. Despite their limitations, these systems can reduce disparities when functioning effectively by supporting the delivery and display of timely and accurate health information at the point of care [36].

In a complementary study, we surveyed 357 patients from the same four clinical sites to examine their preferences for SUD medical record sharing and the factors influencing their decisions. This complementary study focused on patient perspectives using surveys, while the present study explores provider experiences through focus groups, specifically to understand the barriers and facilitators related to HEDIS metrics. Although distinct in scope, both studies highlight common themes, with patients, like providers, identifying SUD stigma as a barrier and provider trust as a key facilitator of data sharing. Stigma was significantly associated with increased sensitivity and reduced willingness to share data, especially with providers outside their primary facility (*p* < 0.001). In contrast, trust in providers and higher satisfaction with care were linked to a greater willingness to share data with all providers (*p* < 0.01) [37].

Both providers and patients in our study expressed concerns about sharing SUD-related information due to perceived risks, which are rooted in real challenges. Potential consequences include confidentiality breaches, stigma, discrimination, legal implications, and fears of law enforcement access. While regulations like 42 CFR Part 2 and HIPAA offer privacy protections, inconsistencies in their interpretation contribute to uncertainty and reluctance to share data. These findings underscore the critical role of stigma and trust in equitable data sharing, improved care delivery, and HEDIS performance. The complementary study has been submitted for publication and is currently under revision.

## 5. Implications

Stigma-related concerns, including anticipated judgment from healthcare providers, deter patients from sharing sensitive data. Providers will benefit from stigma-reduction initiatives as well as assistance in navigating privacy laws and enhanced approaches to handling sensitive information. Such approaches can improve confidence in data sharing and reduce errors or delays.

Moreover, enhancing data-sharing practices can directly support improved care quality as performance on HEDIS metrics related to care coordination, transitions, and follow-up care [38,39]. Our study emphasizes the need for better integration between behavioral health and general healthcare providers. HEDIS measures, such as Initiation and Engagement of Alcohol and Other Drug Abuse or Dependence Treatment (IET), rely on effective coordination among providers. Improved data-sharing practices, supported by optimized health information systems, can reduce delays in treatment and improve engagement rates, positively impacting these metrics.

Transparent and effective communication, as highlighted in the study, not only facilitates data sharing but also strengthens trust and rapport between providers and patients. This aligns with HEDIS measures that assess experience of care, such as care coordination.

The complexity of state and federal laws such as 42 CFR Part 2 create significant barriers to data sharing. Policymakers should consider revising these regulations and related rules to balance patient confidentiality with the need for seamless care coordination and provide clear guidelines and examples to reduce provider hesitancy.

### Limitation and Future Direction

The focus groups were conducted in limited clinical settings, which may not fully capture the diversity of provider experiences in other regions or care models. Additionally, the qualitative nature of the study, while rich in detail, may not generalize across broader populations. Longitudinal studies assessing the impact of specific interventions, such as regulatory reforms or technological improvements, on care outcomes would also provide valuable evidence for practice and policy.

## 6. Conclusions

This study captures valuable insights from providers on the barriers—such as legal concerns, stigma, and technical limitations—and facilitators, including trust, patient education, and supportive health information systems, that impact SUD data sharing. Addressing these challenges through stigma-reduction initiatives, policy reforms, and technological advancements is essential for achieving more equitable, integrated, and effective care for individuals with SUD. Additionally, open discussions about patients’ right to share or withhold private data, such as drug test results, and its potential use are essential in building trust.

## Figures and Tables

**Table 1:** A summary of the themes identified as barriers and facilitators to SUD data sharing.

	Themes	Participants Discussing (n)	Percentage (%)
	Patient reluctance to share	15	48%
Barrier	Data access challenges	13	42%
	Poor provider coordination	9	29%
	Incomplete health information	8	26%
	Complexity of SUD laws	7	23%
Facilitator	Patient understanding	8	26%
	Patient–provider relationship	5	16%
	Health information systems	5	16%

## Data Availability

The original contributions presented in this study are included in the article. Further inquiries can be directed to the corresponding author.
